# Exploring the protein-protein interface of integrin-galectin complex for drug development against HIV infection- a bioinformatics study

**DOI:** 10.1186/1471-2334-14-S3-P78

**Published:** 2014-05-27

**Authors:** M Xavier Suresh, R Barani Kumar, V Sravani, P Suma Priya

**Affiliations:** 1 Department of Bioinformatics, Sathyabama University, Chennai – 600119, India

## Background

The formation of protein-protein complexes of the transmembrane receptor integrin and galactoside binding protein galectin assist in adhesion by bridging between cells or cells and the extracellular matrix and form adhesive networks. When HIV is present the galectin bridges between the CD4 co receptor and gp120 ligands thus facilitating HIV infection of the T cell. The main aim of this study is to investigate the interaction of galectin-3 with glycoligands, integrin, natural compound curcumin and its analogs.

## Methods

The structures of proteins were obtained from PDB whereas natural compound curcumin and their analogs have been fetched from PubChem and Drug Bank databases. ZDOCK, FireDock algorithms were used to predict the protein-protein complex and molecular docking studies were carried out using Hex8 and Discovery Studio.

## Results

Analysis of the results reveals that the glycoligand 3-αGal-LacNAc was found to bind with the CRD of galectin-3 showing interactions with amino acids GLN150, LYS176 and TRP181. The curcumin analog 11 was found to bind with amino acids like ARG144, ARG162, SER237, GLU184 and ASN119 which forms part of CRD of galectin-3. Analysis of the docking poses of protein-protein complexes reveals that the CRD of galectin-3 is the most probable binding interface for integrin molecule.

## Conclusion

The knowledge of the mechanism of interaction between integrin and galectin may provide important therapeutic opportunities and the proposed compounds may act as a scaffold for drug design against HIV infection.

